# Experiences of family carers supporting older people within the last year of life in rural and remote areas in the UK

**DOI:** 10.1093/ageing/afae169

**Published:** 2024-08-09

**Authors:** Caroline Mogan, Nathan Davies, Karen Harrison-Dening, Mari Lloyd-Williams

**Affiliations:** Liverpool John Moores University, Faculty of Health, Tithebarn Street, Liverpool L2 2ER, UK; Research Department of Primary Care and Population Health, University College London, Royal Free Campus, Rowland Hill Street, London NW3 2PF, UK; Dementia UK, Research & Publications, One Aldgate, London EC3N 1RE, UK; Liverpool John Moores University, Faculty of Health, Tithebarn Street, Liverpool L2 2ER, UK

**Keywords:** rural healthcare, end-of-life, family carers, palliative care, qualitative research, older people

## Abstract

**Background:**

In the UK, a large proportion of older adults live in rural/remote locations. More people are dying at home and require care from their families. Little is known about the experiences of family carers of older people in rural/remote areas in the last year of life.

**Aim:**

To explore the experiences of current and bereaved family carers who support/ed an older person in a rural area in the UK towards the end-of-life.

**Design:**

Qualitative methodology using semi-structured interviews and reflexive thematic analysis methods.

**Method:**

Interviews were conducted with family carers of rural/remote-dwelling older people in the last year of life. Participants were recruited through national support services, third sector organisations and social media.

**Results:**

Interviews were conducted with 20 family carers. Most were female (*n* = 17) and aged 52–80 years. Family carers experienced difficulties in accessing health and social care in rural/remote areas due to workforce and skills shortages within their regions. The wider community helped with practical tasks and made carers feel less alone. Community-based services, such as day care, helped to provide respite for carers and promoted meaningful activity and social inclusion for older people. Although internet access was problematic, family carers gained support remotely via social media and telehealth services.

**Conclusion:**

Family carers of older people in the last year of life in rural/remote areas value support from the wider community. Further work is required to understand how Public Health approaches to palliative care and workforce distribution can support rural/remote carers and older people.

## Key Points

- Workforce and skills shortages in rural areas lead to older people not receiving necessary care in the last year of life.
- Ageing in place and being able to communicate in one’s own language are critical elements of good care for rural older people.
- Rural communities can offer valuable support to family carers, including assistance with daily tasks and social connection.

## Background

Rural UK areas are very diverse, ranging from open countryside with a scattering of small towns and villages to coastal communities dependent on fishing or tourism, former mining areas and commuter villages [1]. Across the UK, there is no single definition of rural areas. The Office for National Statistics defines rural in England and Wales as settlements of <10 000 usual residents [2]. Whereas in Northern Ireland, the default definition of rural is settlements of <5000 population [3] and in Scotland it is 3000 [4].

Rural and remote areas have a higher proportion of older residents, and rural communities are becoming increasingly older [5]. Between 2001 and 2015, the population aged 65 and over increased by 37% in rural areas compared to only 17% in urban areas [6]. It is estimated that by 2039, nearly half of all households in rural areas will contain people aged 65 or over [5]. As life expectancy increases, so too does the prevalence of complex chronic illnesses and the challenge of providing integrated palliative and end-of-life care. While the UK has previously been ranked as having the best quality of death and providing a high quality of care [7], this experience has often varied between patients [8], especially those living in more rural and remote areas [9].

In the UK, palliative care in rural areas generally follows the same principles as in urban areas, and models of service delivery include inpatient palliative care units and hospices, multidisciplinary consultation teams (at home, in the community and in hospitals), day care facilities and outpatient services. Palliative and end-of-life care services also frequently work with disease specific charities and local voluntary groups when caring for older people at the end of life. However, when compared to those living in urban areas, older people in rural areas face additional barriers to accessing these services due to long travel distances, a lack of public transport, physical and social isolation, limited out-of-hours services and limited staff resources and specialist services [10]. Additionally, lower population density in rural and remote areas means that it can be more difficult and expensive to create and maintain comprehensive service infrastructures. Consequently, rural and remote areas can be disadvantaged in terms of access to services and activities, and this can exacerbate risks of social isolation and reduced mobility and result in older adults lacking adequate support and health care [11].

The existing research highlights the major inequities for those living in rural areas of limited access to palliative and end-of-life care. People living in rural areas are under-represented in hospice populations [12] and less likely to die in a hospice [13]. Rural day care centres have more older people with cancer than urban day care centres [14], suggesting that distance from palliative care provision and hospice day services causes inequity of access to palliative care. Coupled with the lack of social care within rural areas [15], this is a major area of concern affecting all four nations. Globally, the proportion of people dying at home is increasing. A recent study [16] examining trends in place of death for adults in 32 countries, found that overall, the percentage of home deaths rose from 30.1% in 2012–2013 to 30.9% in 2018–2019 (before the pandemic) and further to 32.2% during the pandemic (2020–2021).

In England, home deaths increased from 18% of deaths in 2004 to 24% in 2019 [17]. The pandemic, however, rapidly accelerated this trend, with three more people dying at home than the pre-pandemic five-year average. In 2020, deaths at home among people aged 85 years and over were 43% above average in England and 38% above average in Wales [18]. A total of 26 730 people in Scotland died at home or outside hospital/care setting, which was 7826 more deaths compared to the five-year average. In Northern Ireland, 71% of deaths occurred at home. While this may be in line with longstanding policy ambitions for more people to die in their usual place of residence [19], The King’s Fund reported that for many of these deaths there may have been inadequate symptom control and inadequate social care [20].

The large increase in home deaths does not appear to be declining, suggesting that more people will be dying at home in the future. With increasing numbers of older and frailer people in the population [21], the informal care provided by family and friends plays a vital role in supporting older people at home as their health deteriorates and end-of-life approaches [22, 23].

Exploration of family carer experiences offers valuable insights into how health and social care services provide care and support to families at the end-of-life and can aid our understanding about how they can be improved in the future. A systematic review [24] of 11 studies exploring rural end-of-life care experiences of family carers found that barriers to care included limited access to health care professionals with palliative care training, paid in-home carers, after hours pharmacy, pain relief and respite care. However, this review was not restricted to studies on older people, and only three of the studies reviewed included people from the UK. A recent qualitative study [25] with 15 rural family carers of older people in the USA found that inadequate access and lack of resources in rural areas cause pain and distress for both older people and their families. They suggested that initiating early palliative care models, improving telehealth communication skills, collaborating with community partners and using culturally congruent care delivery approaches can improve care for older people in rural communities.

Articulation of the rural voice in palliative and end-of-life care is increasing, particularly in countries, such as the USA and Australia. However, there are still a limited number of published studies reporting family carers’ experiences and perspectives from within rural and remote communities in the UK. The aim of this study was to explore the experiences of current and bereaved family carers who have supported an older person in a rural and/or remote area in the UK towards the end-of-life.

## Methods

### Design

This was a qualitative study based on constructivist epistemology using semi-structured interviews, analysed using reflexive thematic analysis [26]. The constructivist lens allowed for contextual understanding of carer experiences by paying close attention to the social, cultural and historical factors that influenced their perspectives.

### Sample and recruitment

Purposive sampling supplemented with snowballing techniques [27] was used to identify current and bereaved family carers from the UK who support/ed a rural-dwelling older person towards the end-of-life.

Rurality was defined in England and Wales using the ONS Urban Rural Classification eight-point scale [2]. In Scotland, it was defined using the Scottish Government Urban Rural Classification [4], and in Northern Ireland, it was defined using the Northern Ireland Statistics and Research Agency Urban Rural Classification [3]. We also used the United Nation’s definition of older persons as those aged 60 years or over [28].

We aimed to incorporate a range of characteristics such as gender, palliative condition, location and carer relationship. A number of national support services and third-sector organisations were contacted, including hospices, palliative care services, carer groups, day care centres as well as rurally situated palliative and disease-specific charities. All services we approached agreed to provide information about the study either in newsletters, on social media channels, or they directly contacted eligible participants to discuss the study. Contact details of those who were interested were forwarded to a researcher, who then contacted the participants. Appeals for participants were also made on local radio stations and in newspapers.

All participants were offered a £20 gift voucher following their interview. However, this was not stipulated within the study recruitment material and the participant information sheet, as this could have been perceived as coercion.

### Data collection

Interviews were conducted between November 2022 and April 2023 by one of the authors (C.M.), who is an experienced researcher in palliative and end-of-life care. Interviews were guided by an interview schedule, which was informed by relevant literature [23, 24]. The interview schedule was piloted with the study’s Patient and Public Involvement (PPI) representative, who is a former family carer.

We also collected demographic data on carer and care recipient age, gender, ethnic background, care recipient postcode [to generate an Index of Multiple Deprivation (IMD) quintiles with 1 being the most deprived and 5 being the least], rural classification, years living in rural location, carer relationship and cause and place of death.

Interviews were conducted either in person, over the telephone or via videoconferencing depending on the participants preference. All interviews were audio-recorded and transcribed verbatim. All data were pseudo-anonymised.

### Analysis

Reflexive thematic analysis was used to analyse data and develop themes [26]. Two members of the research team read all the transcripts (M.L.W. and C.M.), which were imported into Nvivo 14 and coded by CM. Through a series of discussions, initial code lists were refined and initial themes developed. Themes were then refined, while interpretation and meaning of each theme were discussed with the whole team (experienced in dementia care and palliative care), enabling them to contribute to findings and revise iteratively.

### Ethical considerations

Participants were provided with oral and written information about the study. Those who were seen face-to-face provided written informed consent, and those who were interviewed over the phone or via videoconferencing provided oral consent, which was audio recorded. Participants were informed they could stop the interview at any time due to its sensitive topic. However, none expressed a wish to stop an interview or required additional support. Participants were provided with information about support organisations as part of the study debrief.

Ethical approval was obtained from University Research Ethics Committee [Ref: 22/NAH/041].

## Results

Twenty family carers were interviewed. Most were female (*n* = 17) and predominantly adult children of the older person (*n* = 13). Carer ages ranged from 52 to 80 years (mean 64.9). Care recipient ages ranged from 65 to 98 (mean 85.3). Most had lived within their rural area their entire life (*n* = 16), with 1 living within their rural area over 10 years, 2 between 5–10 years and 1 between 0–5 years. Seven carers were recruited following an appeal for participants on a Scottish radio station, 5 via posts on social media, 5 via publicity within a rural day care centre, 2 via publicity sent to cancer charities and 1 from publicity sent to dementia charities. Participant characteristics are reported in Table 1.

**Table 1:** Participant characteristics (*N* = 20).

**Participant**	**Country**	**Rural classification**	**Years living in rural area**	**Carer demographics**			**Cared for demographics**				
				Gender	Age	Relationship	Gender	Age	Deceased	Cause of death	IMD
01	Wales	Rural Village	Lifetime	Male	52	Son	Female	81	Yes	Pneumonia	5
02	England	Rural Village	Lifetime	Female	56	Daughter	Female	81	Yes	COVID-19	1
03	England	Rural Hamlet & Isolated Dwelling	Over 10 years	Female	80	Wife	Male	92	Yes	Alzheimer’s disease	2
04	Scotland	Remote Rural Area	Lifetime	Female	63	Daughter	Female	91	Yes	‘Old Age’ (Undisclosed condition)	4
05	Scotland	Very remote small town	Lifetime	Female	52	Daughter	Male	80	Yes	Colon cancer	3
06	Scotland	Remote Rural Area	Lifetime	Female	64	Daughter	Male	89	Yes	Prostate cancer	4
07	Wales	Rural village in sparse setting	Lifetime	Female	78	Wife	Male	88	Yes	Alzheimer’s disease	4
08	Wales	Rural town and fringe in a sparse setting	5–10 years	Female	65	Wife	Male	70	Yes	Cancer	5
09	Wales	Rural town and fringe	Lifetime	Male	59	Son	Female	87	Yes	Vascular dementia	5
10	Wales	Rural village	Lifetime	Female	59	Daughter-in-Law	Female	91	Yes	Stroke/dementia	3
11	Wales	Rural village	Lifetime	Female	69	Wife	Male	75	No	N/A	4
12	Wales	Rural village in sparse setting	Lifetime	Female	70	Daughter	Female	91	No	N/A	3
13	Scotland	Very remote small town (Island)	Lifetime	Female	67	Daughter	Female	97	No	N/A	4
14	Scotland	Very remote rural area (Island)	5–10 years	Female	71	Wife	Male	65	Yes	Motor neuron disease	4
15	Scotland	Very remote small town (Island)	Lifetime	Female	68	Daughter	Female	91	Yes	Sepsis	4
16	Scotland	Very remote small town (Island)	Lifetime	Female	63	Daughter	Female	86	Yes	Brain tumour	4
17	Scotland	Very remote rural area (Island)	Lifetime	Female	74	Niece	Female	98	Yes	‘Old Age’ (Undisclosed condition)	4
18	Scotland	Very remote rural area (Island)	0–5 years	Male	70	Son	Female	92	Yes	Dementia	4
19	Scotland	Very remote rural area (Island)	Lifetime	Female	58	Daughter	Male	87	Yes	Prostate cancer	3
20	England	Rural village	Lifetime	Female	60	Wife	Male	74	Yes	Parkinson’s disease	4

Despite the diversity of the rural locations under study, family carers reported a range of shared experiences. Five overarching themes were developed: retaining connection to place, accessibility of health and social care, support from the wider community, rural respite services and online support.

### Retaining connection to place

Throughout the interviews, family carers discussed how the older person they supported had wanted to remain at home towards the end of their life, or at least be close to the place where they had lived. Many described the older person as having a strong sense of the rural landscape, as well as the traditions, culture and language of the area that were embedded and central to their being. For family carers, it was important to support the older person to retain these connections towards the end-of-life as they provided a sense of belonging, which was vital to their identity.

#### Connection to the land

Older people appeared to have deep emotional ties or investments in the land where they lived. This was often based on genealogical linkage, memories with friends and family or connection with nature. These attachments were usually described in emotive terms, such as being in a place of ‘happiness’ (P04: Daughter, aged 63, Scotland) or ‘peace and well-being’ (P02: Daughter, aged 56, England).

For others, where the rural land had been a source of sustenance and livelihood, connection was described in a functional sense, and maintaining an understanding of the value of the land, its crops and its animals was important for older people even in the final days of life:

We had cows in a big shed and he would feed them every day well into his eighties and right up until the last moment he was still asking about the farm and he actually died at harvest time so he was asking ‘Have they got the harvest in, how much was the barley worth’… I went out and brought him some barley and straw so that he could smell it.

P06: Daughter, aged 64, Scotland

While rural areas were generally viewed as good places to live, family carers referred to the challenges that older people faced when navigating the landscape and accessing activities that they once enjoyed. One participant who lived on a farm explained how she found it difficult to keep her husband safe as his dementia advanced and he began to wander:

He would insist on going out, but it was too dangerous on the farm, I had to keep all the doors locked, I had to hide the keys and he started trying to go out the windows, I had to buy alarms for the windows, and it was very difficult.

P11: Wife, aged 69, Wales

#### Connection to rural traditions, culture and language

The historical, traditional and cultural aspects of rural life were also important components of the older person’s identity. Many had formed deeply rooted cultural traditions, which family carers felt were important to be upheld towards the end-of-life. One participant spoke about a time that her mother went into a care home for rehabilitation. It was located many miles away from the family home, which they felt was disorientating as she was disconnected from her usual way of living:

The [care home] was on the coast, north coast, I think there was only one Welsh speaker there and they had no idea of a farming background at all …so she was in a totally different world…the television was always on the English channels, nothing in Welsh or anything like that so it was a really strange world for her, you know things like that, our way of life is so different to many people.

P12: Daughter, aged 70, Wales

She explained how her mother had even stopped eating as the food provided at the care home was so unfamiliar to her:

Sometimes they had KFC as a treat…my mum had no idea what KFC was…she eats every morning what we call “Briwas” which is made up of oats and Bovril and I tried to tell them but they didn’t know how to do it…so then she went down for a while and they were concerned that she wasn’t eating.

P12: Daughter, aged 70, Wales

Even for those older people who had moved to rural and remote areas in their later life, experiencing local traditions and customs could be rewarding and many were proactive in getting involved in community activities and events, which helped them to develop connections with others:

Dad was very popular because he would go to all the festivals, folk festivals, art, science festivals, he would go to all the things in the community, he absolutely loved it and he would eat everywhere, he would be off to all the islands, he was known around, we would walk down the street and everyone would say hello to him.

P19: Daughter, aged 58, Scotland

Many of the older people living in rural areas of Wales spoke Welsh as their first language. While most had been proficient in English as a second language, some family carers explained that their fluency had deteriorated as they became unwell, especially in those who had dementia. This could result in language barriers with health and social care professionals, leaving the older person feeling excluded or misunderstood, which impacted on how supported they felt:

I made loads of complaints, they took her to [hospital], and she was going to the toilet, this nurse came, and [my mum] said, “Aros,” because she wanted privacy, the nurse barged in and I stopped her and said, “She said ‘aros’, stay”, they couldn’t understand Welsh, they couldn’t follow anything, so I was disgusted, because she’s Welsh, there is no communication, there is no being kind, if you talk with my mother, nicely, in Welsh, she’d probably be alright, but there was no one there to do it.

P09: Son, aged 59, Wales

One participant noted how their experience of care was much better received when healthcare professionals acknowledged the importance of language and were able to use even just a few Welsh phrases:

They were not Welsh speaking, but they would try, you know they would say things like “rydym am eich troi rwan” you know “we are going to turn you over now”, they would try, fair play to them, they would, they had phrases in Welsh.

P10: Daughter-in-Law, aged 59, Wales

### Accessibility of health and social care

Poor accessibility to health and social care services, particularly specialist palliative and end-of-life care, was a theme that ran throughout all interviews. Family carers contributed this mostly to services being situated centrally in, or closer to urban areas. They recognised that this created issues relating to travel distance, transportation and the rural workforce.

#### Travel distances

Distance from health and social care services was identified as a major barrier to receiving health and social care, with most older people having to travel many miles to receive care, particularly for specialist consultations and treatments, such as chemotherapy.

It was a five-hour journey to take him for chemotherapy, I would wait for him to have the treatment and then bring him back home, one time they had discharged him at midnight and I was driving him back, I nearly got him home, and he started having respiratory problems, so I had to drive about forty miles with him hardly breathing, with an ambulance on the phone, I couldn’t stop the car because it was dark, I was on a rural road, I thought my best chance was to get to the hospital, so I got him there and then I slept in the car.

P08: Wife, aged 65, Wales

Family carers also expressed concern that because of the distance to hospitals, the older person may have delayed or avoided specialty care because of the challenges it created:

I realised dad wasn’t get the support he needed from the specialists because of the distance, I hadn’t appreciated how difficult that was for dad until it was too late, it meant that they were much much later starting his chemo treatment and dad had insisted that he didn’t travel up and down to [hospital that was 110 miles away], he wasn’t fit enough to take that journey, I think if things hadn’t gone how they did then he would still be here now, I mean they were never going to cure him but he had that opportunity of having maybe another couple of years decent life, you know, I feel like he had that taken away from him just because he was living in an area where he wasn’t having access to services that couldn’t have made it easier.

P05: Daughter, aged 52, Scotland

Domiciliary care represented a lifeline for family carers, helping them with practical and personal aspects of care that allowed the older person to continue living in their own home. However, family carers observed that the sparsity of the population over such large rural areas meant that distances between the older people’s homes imposed additional expenses for care companies in terms of both time and cost:

There wasn’t anybody who had the capacity to provide two [domiciliary carers] four times a day in this area because it’s a long way to come out for anybody and if they’ve got staff working all over the district it was just too far and too many people for them to supply so nobody could do it, not even the big companies, nobody.

P03: Wife, aged 80, England

For people who lived on the UK islands, it was commonplace for healthcare staff to be flown across from the mainland:

The [palliative care charity] nurses had to come [over to the island] from [the mainland] because we didn’t actually have any [palliative care charity] nurses here, they have one or two that will do overnights for people but I think that they also have to work at the [general hospital], so I think that there is a severe lack of nursing hours for that as well yes, the [palliative care charity] e nurse came from the mainland for the last two nights and she had to be put up in the local hotel.

P16: Daughter, aged 63, Scotland

#### Transportation

Issues of transportation also featured significantly in the interviews. Some older family carers did not have access to transport or were no longer able to drive because of age or health status. Many older people relied on family or friends to take them to appointments, but others lacked social support to request assistance for time-consuming journeys, relying instead on taxis, which could be expensive and unsuitable:

They had her on dialysis right up until she died but I think that was quite traumatic because of the length of the journey she had to travel for it, she was travelling from the depths of [the county] right up to [nearest town] for her dialysis so it was at least an hour, an hour and a half, she had inner ear problems so she would vomit for most of the journey but there were just no local facilities, and it seemed to me that there was nobody local who properly understood what care she needed so she was in difficulties, we had to go a long way to get help.

P02: Daughter, aged 56, England

While voluntary bus services were available in some areas, some had difficulty reaching pick-up locations, and the long journeys could be uncomfortable:

It was really difficult for dad to get to appointments at [the hospital] because often we weren’t able to drive him there and he wasn’t well enough to take the NHS transport, I mean it’s great it’s there but it’s a minibus, it’s very uncomfortable and it often goes from village to village so it can make what should be a two hour journey into a four hour journey because you’re stopping to drop off and pick up other sick people…yeah the distance became a huge huge issue and it definitely impacted on my dad’s recovery and on the way I could support him because I couldn’t drive him and they have [a cancer charity] in [nearest large town] and that’s all palliative and supportive care for cancer patients but we could only access that over the phone, we couldn’t drive Dad all that way down there, it’s 100 miles.

P05: Daughter, aged 52, Scotland

Even with adequate transport, the condition of rural road networks could present difficulties at specific times of the year, with isolated roads experiencing severe conditions in winter. This could interfere with travel or cut people off entirely:

The weather can change so drastically here [one time] we got stuck, but [my elderly husband] couldn’t even walk a hundred yards so we just sat there and eventually a snow plough came past and then a four wheel drive jeep stopped and said “I can give you a tow” so he drove us up and he left us at the top to get down the other side, then we went to turn into our road but obviously there’s no snow plough gone down there so we were stuck again so I got out and left [my husband in the car] to walk to a farm to get help.

P03: Wife, aged 80, England

#### Workforce issues

Some family carers associated poor accessibility to services with workforce issues, such as difficulties of rural staff recruitment and retention. Discussions about insufficient numbers of health professionals included inadequate work contracts in rural and remote areas and a lack of low-cost housing meaning younger people were less likely to return to work and live in the rural area they grew up in. This was an issue for younger people generally wanting to work or live in rural areas:

A lot of young people go away they do their training and then they end up staying wherever you know they meet somebody or whatever and they stay away and those that do come back struggle to get jobs, to get a contract because NHS don’t seem to want to give you know permanent contracts to people.

P16: Daughter, aged 63, Scotland

Particularly in the face of increasing older age profiles in rural areas, family carers believed that the shortage of specialist palliative care professionals meant that services could not cope with demand and needed to use their expertise and experience to triage:

The district nurse said that the [cancer charity] nurses were short staffed and the story that she had told me was that the [cancer charity] nurse had to choose between three families that day who needed the palliative or end-of-life care and the [cancer charity] nurse had to decide which family needed more than the other.

P05: Daughter, aged 52, Scotland

In some areas, a lack of permanent rural health and social care workers could result in the use of agency staff:

But unfortunately, the other side of that is there are few choices, care is in an absolute crisis here in Scotland, there aren’t the people around to do it, there’s a lot of agency staff being used locally which has never been heard of before say thirty years ago it was never heard of. They have to have agency staff to cover but agency staff are being paid a lot more money and that’s causing the local staff a lot of stress.

P13: Daughter, aged 67, Scotland

Other workforce issues included a tension between specialist and generalist practice and the inherent de-skilling this created in some areas. In one area, e.g. a specialist palliative care unit had closed following the retirement of several nurses. The remaining nurses were moved to the general hospital, and because of staffing issues, they would be asked to work in other areas. This resulted in end-of-life care being delivered on general wards:

There was three of us older [specialist] nurses who retired in 2020 and they spread all the rest, the [specialist] staff through the hospital and they’ve just got sucked into the other wards because they’ve been so badly staffed, they are never going to make it the way it was before. We had dedicated ward staff for [the specialist unit] and that will never happen again, end-of-life care is now done in inpatients two.

P16: Daughter, aged 63, Scotland

The loss of this service came as a great shame to one participant whose father had benefitted from it before his death in January 2020:

[The specialist nurses] made it so special for him, massaging him, talking to him, talking to us and I was just holding his hand and it was just you know, I can’t fault their care whatsoever for him or myself really….so, it was such a beautiful part I mean it’s exactly the same as the hospital, you had the hospital just around the corner, they worked very closely with the hospital it was brilliant, so I think that’s a real, real shame and I don’t know if its lack of funding or I think COVID has changed an awful lot of things though hasn’t it.

P19: Daughter, aged 58, Scotland

Some family carers also expressed a perceived lack of palliative care skills among rural healthcare workers, which they believed resulted in symptoms and signs of deterioration being overlooked:

*I just don’t think the funding is there for the rural services and especially in a county like [this one], it’s the biggest county with the smallest population so there just isn’t that funding for outreach, for community services, so we were just getting [healthcare professionals] who were jack of all trades rather than specialists in what they were doing, I was desperately worried at one point that the community person who was looking after mums care had no real understanding of what her needs were or to identify when she was deteriorating, you know the things to look out for, that worried me, I think in rural areas they miss out on that real specialist care which may save their lives, I wonder if mum had some specialist input at the right time and identified her when she was deteriorating, it might have been a different outcome.*

P02: Daughter, aged 56, England

### Support from the wider community

Many family carers reported that the older person lived within a supportive rural community consisting of nearby family, friends or neighbours who were able to provide practical and emotional support. The sense of community and mutual assistance created a strong foundation for people to look out for one another:

She lived in a community where everyone around her, had all pretty much moved in at the same time, so they were all in their eighties…they would phone each other and the immediate neighbour, she was more able so she could go out and get the prescription and things like that and they got on well, they had created that friendship because they were both Welsh speakers.

P01: Son, aged 52, Wales

Family carers also discussed how the relationships with people they had employed from the local community, such as cleaners and gardeners, were helpful. Not necessarily because of the tangible assistance they had been paid to provide but by offering social and emotional support. Many were seen to adopt a ‘dual role’ and went above and beyond the duty:

And my gardener and his wife were wonderful because I’d been going out for walks with them and then we’d go for a tea and a scone as a takeaway as you could sit outside, we were still in COVID so we started going to the café and getting the scone and the tea and we would sit outside and it was like you know a little bit of respite from caring anyway, it was due to them that I even got out of the four walls.

P03: Wife, aged 80, England

Friends and neighbours could also help family carers and older people stay connected to the wider community by informing them of local events, activities and resources:

Maybe that’s quite arrogant but I automatically expected support from my community [after my husband died], the little community round here is fantastic, the people are so kind, there’s been this ladies badminton group and they’re a hoot, last night there was a bit of a dinner out and how cute is that, you know its things like that and people invite you along for stuff “do you want to go to the pictures”, if you want to do this or do you want to do that, so, I don’t feel isolated, I feel yeah its lonely at times, of course it’s lonely, but that’s the nature of it.

P14: Wife, aged 71, Scotland

Support from the wider rural community was also often reciprocal, with those who had previously been involved in contributing to their local areas receiving lots of support from that community. However, one carer observed that the close support she enjoyed may not be experienced in the future, due to changes in village life norms, highlighting that the rural advantage of closeknit communities may not be accessible for new people moving in:

I do have community support, it’s been a fantastic community in the past, when people were able we ran the Village society and there was always something going on and people loved it, we did that for thirty years but people have died, people have moved and people have moved in an aren’t as interested in the villagey stuff…you get people who move in who don’t want to be a part of the village, they just keep themselves to themselves.

P03: Wife, aged 80, England

Other family carers, reported a lack of community support due to social estrangement caused by geographic isolation, or an attitude of stoicism and self-sufficiency preventing older people from asking for help:

He didn’t want any outside involvement, you know, he was a very proud man, a typical farmer really, he didn’t want anybody to know that his wife had dementia, you know he was very private and very guarded, you know he didn’t want anybody to know really, so it was very difficult.

P10: Daughter-in-Law, aged 59, Wales

Self-reliance among families could also mean that older people became isolated as other members died or moved away:

My mum had seven siblings and my father had lots of siblings, so they were very much a family who relied on family rather than friends and they all lived very close to one another but unfortunately she was one of the last of them, so the rest of them had died so she became really quite socially isolated so she was just relying on us to visit but we lived all over, some of us abroad.

P02: Daughter, aged 56, England

### Rural respite services

For some family carers, looking after the older person could become physically and emotionally demanding, leading to stress and exhaustion. Consequently, respite services, such as day care and in-home respite, were cited as essential supports, which provided family carers with some time away from their care responsibilities.

#### Day care

Day care services in rural areas were valued by family carers and older people, many of whom attended until the final months and sometimes weeks of their life. Not only did day care provide essential respite for family carers but also provided older people with opportunities for social interaction. As family carers could spend considerable amounts of time travelling to different services, day care services that offered transportation were highly appreciated, further alleviating carer burden:

The day care was very good, very, very helpful, it gave me some time and he was quite happy going there, it’s not as though I had to force him to do it, he was quite happy, he had transport from door to door, so it was no trouble to me really. Yes, I was very, very grateful for that.

P11: Wife, aged 69, Wales

Family carers were also pleased that some day care services offered activities that were meaningful and helped connect older people to their rural heritage and language:

She really picked up and it was a pleasure to take her there and the people there so kind and what was best for mum was that they were Welsh speaking and from a rural background knowing about her background of farming and you know the Welsh culture, she played the organ in a chapel for fifty years and things like this you know and they were aware of things like that.

P12: Daughter, aged 70, Wales

Some day care centres also provided meals, ensuring that the older person had access to a nutritious diet, which can be especially important in more remote areas where there may be limited access to supermarkets and fresh produce. Other centres also offered much-needed personal care, which enabled the older person to remain at home for longer. One participant who looked after her husband with dementia explained how, while she had mostly coped well at home, she struggled to get her husband into the bath. However, day care services provided facilities to do this:

In previous years at least two people in the village had been able to go to the day centre where they would give them a bath, wash their hair, give them a meal and they’d do activities and then they’d fetch them back, well that closed, the funding was withdrawn.

P03: Wife, aged 80, England

After the service was withdrawn, the family carer was left to manage alone, which soon resulted in an accident, where her husband slipped and did not retain mobility again:

He fell in the bathroom and he was on the floor for about three hours before the ambulance came and he’d broken his hip, it was quite cold in there because it’s straight onto concrete floor so I was having to get blankets to put underneath him and getting the fan heater on him so when they arrived they could tell straightaway he’d broken his hip, so that was the start of the end really.

P03: Wife, aged 80, England

#### In-home respite

In-home respite appeared to be a favourable option, but availability was often sparse, and it could be very expensive:

Some respite care in the house would have been nice you know, if somebody could have come in and sat with her you know. If we wanted to do something together as a family, we couldn’t you know we couldn’t go as a family anywhere really, you know.

P10: Daughter-in-Law, aged 59, Wales

Some family carers mentioned that they had been directed to local, voluntary agencies who provide a ‘sitting service’ during the day. However, family carers found that these were often restricted to just a few hours per week and so could not fully meet their needs.

Family carers also mentioned that some palliative care charities offered ‘night sits’, allowing them to get some much-needed sleep when caring for the older person at home, yet many others were not aware that these services existed, and in some areas, they were not available at all:

I didn’t need them but I know that other families would have benefited from having to get some sleep so that somebody else is sitting there if you haven’t got anybody else to sit there but there are no [palliative care charity] nurses in [name of Island], or they said that they could apply for somebody to come from [the mainland] blah blah blah, it all seemed a bit of a faff, but maybe that was because of lockdown too but at the moment, there doesn’t seem to be anybody who can help support terminally ill people at home…to have somebody take over to allow you to sleep makes all the difference.

P14: Wife, aged 71, Scotland

### Online support

Family carers spoke about the use of online support from healthcare providers, as well as from friends and family. They talked about how, since the COVID pandemic, many services had moved online. In some ways, this was advantageous for them, as they could access different types of support without having to travel long distances. This could help improve their wellbeing and quality of life:

*I joined Facebook and it was quite good really as I was could talk to my grandchildren, so I’m now connected with them which I wasn’t before, then [I went to a] choir three times a week… and it was really good fun…and I found a [carers group]…and I went on an eight week mindfulness course, manual handling, all online…in the carers group you could chat to the others…I used to talk to my University friends online…they live all over, so we could meet virtually with our coffees…COVID was helpful in a way because for once everybody was in the same boat as me not being able to go out (laughs).*

P03: Wife, aged 80, England

While many welcomed the introduction of telecare and online healthcare consultations and agreed that it was a sign of progress, some reported on how a failure to deliver telecare appropriately could have negative repercussions on older people’s understanding of their treatment plan and subsequently on their health:

In a technical world, there should be more appointments online, but in my dad’s case it was a complete disaster and impacted on his recovery, he didn’t stand the best chance because I had been leaving him to have his Zoom oncology appointments on his own, I’d say “how did they go” and he’d say “oh yeah fine” that whole stiff upper lip thing, because my dad had capacity it didn’t cross their mind that he might need a representative…it wasn’t until dad started getting really sick that the oncologist requested that I be there, it wasn’t until I went on the videocall I realised what he was having to deal with, I was struggling to understand the oncologist, and I thought “no wonder he is so mixed up about what his care was going to be and making decisions about chemotherapy”, he had information overload and he couldn’t understand any of it, and the other thing, the oncologist was in a room with other colleagues so with NHS regulations she had to wear a mask so she’s on a Zoom call with my eighty year old dad with a mask on, do you know what I feel so guilty about that, if I’d have realised I might have been able to interject early.

P05: Daughter, aged 52, Scotland

Additionally, in rural and remote areas, the infrastructure for internet and mobile phone connectivity can be limited, leading to issues, such as slow internet speeds, unreliable connections and even complete lack of coverage, in some areas. This could also be a barrier to receiving support:

Mobile phone signal and internet access is a problem, I’m home now on the landline on the farm but even when you get into the village, which is 2.5 miles away, there there’s nothing at all on any mobile phone or anything, even though there is fibre optic going straight past the village, they say eventually it will come, you know people take it so much for granted that mobile phones work everywhere don’t they, but they don’t unfortunately.

P12: Daughter, aged 70, Wales

## Discussion

This study explored the experiences of family carers supporting rural/remote dwelling older adults towards the end-of-life and has highlighted how the support needs of rural family carers in the UK may differ to those living in, or closer to, urban areas. Rural family carers expressed a strong need to support older people to stay at home within their rural/remote communities. While many of the older people spent most of their final year of life at home, some were transferred to the hospital in the last few weeks or even days, and subsequently died there. End-of-life care at home ideally involves close collaboration between primary and secondary health care [29], including specialist palliative care and/or hospital care [30]. However, consistent with other rural end-of-life care studies [24], workforce shortages, failures of outreach and skills shortages resulted in shortfalls in care, which affected older people’s ability to stay at home until the end.

In rural areas, 41% of people do not have access to their nearest hospital within an hour’s travel by public transport or walking, compared with 6% of users living in urban areas [31]. This ‘distance decay’ has been associated with poorer outcomes for some patients. For example, a recent Scottish study [32] found that patients living more than 60 minutes from their cancer treatment centre had poorer survival and were significantly more likely to die within 12 months regardless of socio-economic status. A systematic review of studies [33], mainly from USA, Canada and Australia, and including four UK studies, also found that rural dwelling people with cancer had a 5% reduced survival compared to those living in urban areas due to inequity in access to screening and treatment and distance from major cancer centres. Consistent with previous research [13], none of the older people in this study died in a hospice. While some did not have conditions that are generally referred to hospice services (e.g. dementia), this may have also been because of distance to services or reluctance of older people to attend.

Family carers also recognised issues related to the rural health and social workforce. Recruitment and retention of the healthcare workforce is essential to maintain access to services and improve health outcomes of vulnerable populations but is a persistent challenge for rural health and social care services [34]. In areas with a smaller pool of local employees, low pay and/or temporary contracts do not help to attract workers, especially when there may be more attractive alternatives on offer from hospitality or retail sectors.

Another important issue for rural family carers was retaining the older persons connection to ‘place’. In rural areas, where the natural environment and community bonds are often closely intertwined, the connection to land and rural heritage, is a central aspect of an older person identity and sense of belonging [35]. There is an emerging body of work focusing on the role of heritage and culture specific to the rural context, which acknowledges their variability and complexities and how these may affect people’s experience of palliative care [24, 36, 37]. Understanding how these differences impact on the choices people make towards the end-of-life is imperative to improving rural services [38].

In the UK, a number of indigenous minority languages are spoken, reflecting the diversity of its modern-day population. According to the UK Census, there are an estimated 538 000 Welsh speakers living in Wales, representing 17.8% of its total population, with most of these residing in rural areas of the North and West [39]. Gaelic is also recognised as an indigenous language of Scotland and is spoken by around 58 000 people (1.1% of its population) [40]. The Irish language is also the main language of around 6000 people in Northern Ireland (0.3% of its population) [41].

Experiences of social exclusion are common among linguistic minority older adults in the UK, resulting in limitations in health service access, language barriers, longer waiting times and receiving flawed and insufficient health information [42]. Other studies have demonstrated that older Welsh speakers are more likely to revert to using Welsh in later life [43]. Additionally, people who are generally fluent in both languages may temporarily lose their command of English in stressful situations, especially those with dementia [44]. This can impact the accuracy of assessment and quality of ongoing care.

Rural day care services were perceived as particularly important, often serving as community hubs that fostered a sense of belonging and connection to the local community for older adults by providing opportunities for engagement with volunteers and other community members. Not only did day care provide respite for family carers, but it also gave older people the opportunity to engage in meaningful activities that were related to rural life and to converse with others in their minority language. A previous study found that when residents in Welsh care homes received support that was congruent with language and cultural needs, there was an improvement in the care experience and well-being, due to enhanced communication and cultural understanding [45].

The wider rural community also played a crucial role in looking after the older person towards the end-of-life, providing a support network that allowed people to continue living at home. The links between social support and health and well-being have been demonstrated [46, 47], and, increasingly, evidence suggests that engaging communities in tackling health issues leads to better results than leaving it solely to professionals [48]. One of the continuing ‘Ambitions for Palliative and End of Life care’ [49] is to engage the community so that ‘every community is prepared to help’. Consequently, there has increased interest in community development approaches that take a strengths-based approach and seek to make the most of community assets. This should be considered when designing new interventions for older people in rural and remote areas.

Rural family carers also spoke of the benefits of using the internet to receive support. Through social media, video calls and messaging apps, carers and older people could stay connected to family and friends. Since the pandemic, a growing number of older adults have taken up or increased their use of online technology [50]. Online platforms, such as WhatsApp, Facebook and other online communities, can help older people exchange information and resources across a broad social circle and access services for daily living needs and medical care [51–53]. Studies have found that these digital engagements can foster resilience and help to reduce loneliness [54, 55]. However, many of these technologies are reliant on good digital connectivity, leaving behind those in areas without reliable broadband.

In England’s rural areas, 11% of premises are unable to access a broadband connection with a 10 Megabits per second (Mbps) download speed, which industry regulator, Ofcom, considers as a necessary speed for everyday online tasks [56]. In view of many healthcare services now relying on telehealth and virtual consultations to reach some patients, it is of great concern that vulnerable groups could not be accessing the information they need to support their health and quality of life.

### Strengths and limitations

To our knowledge, this is the first study to explore the experiences of family carers in the UK who have supported an older person in a rural/remote location towards the end-of-life. Carers and older people represented a range of ages, socioeconomic status, living circumstances and conditions. Data analysis involved multiple analysts with various backgrounds, including PPI members, and iterative team-based discussions in developing codes and themes. These approaches enhanced the analytical rigour of the study.

A limitation to our sample was an overrepresentation of female, daughter-caring relationships. While females are significantly more likely to provide unpaid care than males in every age group up to 75–79 years, the distribution changes with age, with males from the age of 80 years onwards being significantly more likely to provide unpaid care than females [57]. This limits the comprehensiveness of analyses relating specifically to the male carer experience, which may have brought different perspectives to this research.

Future research should seek to recruit a sufficiently large sample of male carers, specifically, including sons, to better understand the needs of this cohort. We also recruited mainly participants who had always lived in rural locations. However, many older people retire to remote and rural locations and hence may have fewer social networks or be less embedded within the local community, thus affecting their support and experience.

Additionally, the study recruited only a limited number of participants from England and none from Northern Ireland, despite our study recruitment materials being widely and extensively shared via local contacts and social media across the four nations. While palliative and end-of-life care services share similarities across the UK, local context may have influenced the findings, and therefore, we may not have been able to give a comprehensive representation of informal carer experiences across the four nations, which would allow between nation comparisons.

Finally, our sample contained only a white population who were all able to speak English, albeit some as a second language. Consequently, the impact of some cultural values on people’s experience and preferences could not be evaluated.

### Future research

More research is needed on rural and remote populations in the UK to evaluate the types of palliative care services provided to older people and the quality of such services. It is of note that hospices were not mentioned within any of the interviews. Consequently, more research is needed to ascertain how these services can support people in rural and remote locations in the UK. We also recommend further studies that focus on the perspectives of different groups of health and social care professionals to understand their experience of delivering palliative and end-of-life care in rural and remote areas. In addition, more research is required to explore the end-of-life preferences of older people in rural and remote locations.

## Conclusion

Family carers in rural and remote areas experience significant difficulties with accessing health and social care for the older people they care for within the last year of life. However, rural and remote communities can offer valuable support to family carers in several meaningful ways, including assistance with daily tasks and social connection. There is increased interest in Public Health and community development approaches to palliative care, such as ‘Compassionate Communities’ that take a strengths-based approach and seek to make the most of community assets. Such initiatives are often reflective of rural communities [58] and may provide practical and cost-effective solutions by complementing existing services to ensure that older people in rural and remote communities, and their family carers, receive the additional practical and emotional support they need towards the end-of-life. Rural family carers could also be supported by being trained on how to deliver basic nursing tasks and how to administer end-of-life medications. However, it must be noted that rural issues are different from urban issues, and this is important to consider when co-designing and organising future services and interventions.
